# The effects of L-carnitine supplementation on lipid concentrations inpatients with type 2 diabetes: A systematic review and meta-analysis of randomized clinical trials

**DOI:** 10.34172/jcvtr.2020.43

**Published:** 2020-09-07

**Authors:** Omid Asbaghi, Sara Kashkooli, Mohammad Reza Amini, Hossein Shahinfar, Kurosh Djafarian, Cain C.T. Clark, Sakineh Shab-Bidar

**Affiliations:** ^1^Student Research Committee, Lorestan University of Medical Sciences, Khorramabad, Iran; ^2^Nutritional Health Research Center, Lorestan University of Medical Sciences, Khorramabad, Iran; ^3^Department of Community Nutrition, School of Nutritional Sciences and Dietetics, Tehran University of Medical Sciences, Tehran, Iran; ^4^Students’ Scientific Research Center (SSRC), Tehran University of Medical Sciences (TUMS), Tehran, Iran; ^5^Department of Clinical Nutrition, School of Nutritional Sciences and Dietetics, Tehran University of Medical Sciences, Tehran, Iran; ^6^Centre for Intelligent Healthcare, Coventry University, Coventry, CV15FB, UK

**Keywords:** L-Carnitine, Lipid Profile, Meta-Analysis, Randomized Controlled Trials

## Abstract

This meta-analysis was performed to assess the effect of L-carnitine supplementation on lipid profile. A systematic search were conducted in PubMed and Scopus to identify randomized clinical trials (RCTs) which evaluated the effects of L-carnitine on lipid profile. Pooled effect sizes were measured using random-effect model (Dersimonian-Laird). Meta-analysis showed that L-carnitine supplementation significantly reduced total cholesterol (TC) (weighted mean difference [WMD]: -8.17 mg/dL; 95% CI,-14.68 to -1.65, I^2^=52.2%, *P *= 0.041). Baseline level of TC was a source of heterogeneity, with a greater effect in studies with a baseline level of more than 200 mg/d (WMD: -11.93 mg/dL; 95% CI, -20.80 to-3.05). L-carnitine also significantly decreased low-density lipoprotein-cholesterol (LDL-C) (WMD:-5.22 mg/dL; 95% CI, -9.54 to -0.91, I^2^=66.7%, *P *= 0.010), and LDL-C level <100 mg/dL), trial duration,and L-carnitine dosage were potential sources of heterogeneity. L-carnitine supplementation appeared to have no significant effect on high-density lipoprotein-cholesterol (HDL-C) (WMD: -0.51 mg/dL;95% CI, -2.45 to 1.44) and triglyceride (TG) (WMD: 2.80 mg/dL; 95% CI, -8.09 to 13.69). This meta-analysisrevealed that L-carnitine may have favorable effects on lipid profile, especially LDL-C and TC. However, further RCTs are needed to confirm the veracity of these results, particularly among hyperlipidemic patients.

## Introduction

Diabetes mellitus is a common human metabolic diseases in all countries, and of all incidences of diabetes; type 2 diabetes is the most common, comprising about 90% of patients.^1^ Type 2 diabetes is a major health concern and often occurs concurrently with dyslipidemia in individuals at high risk of cardiovascular disease.^2^ Indeed, dyslipidemia, which is defined as abnormal level of serum lipids including decreased level of high-density lipoprotein-cholesterol (HDL-C), and elevated level of plasma triglyceride, cholesterol, and low-density lipoprotein-cholesterol (LDL-C) particles, is a key risk factor for cardiovascular disease in people with diabetes.^3,4^ Several other factors unrelated to glycemia or insulin resistance like chronic kidney disease, hyperthyroidism and genetic disorders of lipoproteins, may influence lipid profile. Unhealthy diet including high fat and low fiber diets, elevated weigh, lack of glycemic control, and smoking also are associated with increased risk of dyslipidemia in type 2 diabetes. Accumulating evidence has suggested potentially beneficial effects of functional foods for dyslipidemia, which subsequently could ameliorate the long-term diabetes-related complications, including macro- and microvascular diseases.^5^

L-carnitine (3-hydroxy-4-N-trimethylammonio-butanoate) is a water-soluble, unbound, amine that plays an important role in transportation of long-chain fatty acids into the mitochondria, therein facilitating an increase in fatty acids metabolism.^6-8^ L-carnitine is synthesized endogenously from lysine and methionine or can be obtained from animal products, such as milk and meat.^9^ Moreover, L-carnitine might change glucose catabolism because it acts as a carrier of acetate from mitochondria to the cytoplasm, which results in reducing the acetyl CoA/CoA ratio in mitochondria, increasing the activity of pyruvate dehydrogenase.^10-12^ Indeed, previous studies have shown that treatment with L-carnitine may has favorable effects in the management of diabetes, insulin resistance, elevated blood pressure, and dyslipidemia.^13-16^ However, in contrast, other studies have indicated that L-carnitine may increase fasting triglyceride in patients with type 2 diabetic.^17^

Although previous randomized clinical trials (RCTs) have assessed the effect of L-carnitine in the management of dyslipidemia in adults, the current evidence base is equivocal.^16-25^ Accordingly, this systematic review and meta-analysis was conducted to investigate the effects of L-carnitine supplementation on lipid profile in patients with type 2 diabetes.

## Materials and Methods

### Search strategy and study selection

We conducted a comprehensive search in PubMed and Scopus to find English published papers. We used the medical subject headings (MeSH) and non-MeSH terms keywords: (“Vitamin BT” OR L-carnitine OR “L carnitine” OR carnitine OR levocarnitine OR bicarnesine OR L-acetylcarnitine OR acetyl-L-carnitine) OR (Triglyceride OR Triacylglycerol OR TG OR cholesterol OR Lipoprotein OR “very low density lipoprotein” OR VLDL OR “low density lipoprotein” OR LDL OR LDL-C OR “high density lipoprotein” OR HDL OR HDL-C OR “lipid profile”) OR (Diabetes OR “type 2 diabetes” OR T2D OR diabetes mellitus OR T2DM). Moreover, manual searching was carried out to find relevant studies from reference list of the eligible papers. We conducted all searches from database inception to 18 January 2020. In addition, to ensure we found any other relevant trials, all reference lists of included RCTs, previous review studies and trial were searched. The search keywords and strategies were applied according to the PICOS model which includes question about patient (patients with T2DM), intervention (L-carnitine supplementation), comparator (placebo), outcome (triglyceride, TG; total cholesterol, TC; low-density lipoprotein-cholesterol, LDL-C; and high-density lipoprotein-cholesterol, HDL-C), and study design (parallel and cross-over clinical trial). We included studies if they met the following criteria: (A) RCTs comparing the effect of L-carnitine supplementation to placebo; (B) involved patients with T2D; (C) reported lipid profile such as, TG, TC, LDL-C and HDL-C; and (D) treatment duration of at least 4 weeks. Two (OA, SK) authors independently evaluated the publications based on the titles and abstracts, and any disagreement was resolved through discussion.

### Data extraction

Two reviewers (OA, SK) independently obtained the data for this meta-analysis and evaluated the quality of eligible studies, whilst in instances of disagreement, a senior author was involved to achieve consensus. The following data were extracted into a standardized form: (1) characteristics of studies including study design, authors information, year, country, trial length, and the number of trial arms; (2) subjects’ information, including inclusion criteria, age, sex, and health status; (3) outcomes assessed, including baseline and final values of lipid profile including LDL, HDL, TG, and TC.

### Quality assessment

The Cochrane Risk of Bias Tool was applied to assess the studies’ quality.^26^ This tool uses five methodological domains focusing on bias arising from selection, randomization, blinding, missing data and measurement of the outcome to categorize individual studies as low risk, high risk, or unclear risk of bias.

### Statistical analysis

Meta-analysis was carried out using Stata software, version 12.0 (StataCorp LP, College Station, TX, USA). We investigated the L-carnitine effects on lipid profile by pooling mean and standard deviation (SD) values of the baseline and the end of the studies in both intervention and control groups. In the absence of mean and SD, we converted the other reported data of variation into mean and SD by using proper formula.^27^

A correlation coefficient of 0.8 was considered as R-value of the above-mentioned formula.^28^ The pooled data were computed using weighted mean difference (WMD) with 95% confidence interval (CI). The heterogeneity was assessed using the Cochrane’s test and *P* value *<*0.1 was defined as significant. In case of significant heterogeneity, the DerSimonian and Laird random- effects model was applied. Subgroup analysis based on baseline serum lipids, trial duration (12 ≥ weeks > 12), and L-carnitine dosage (less than 3000 mg/dL or ≥3000 mg/dL), was performed to find the source of heterogeneity. The results were considered statistically significant at the level of 0.05.^26^ Sensitivity analysis was performed to estimate the possible source and effect of the bias, and publication bias was evaluated using Egger’s test.^29^

## Results

### Selected studies

**Figure 1:**
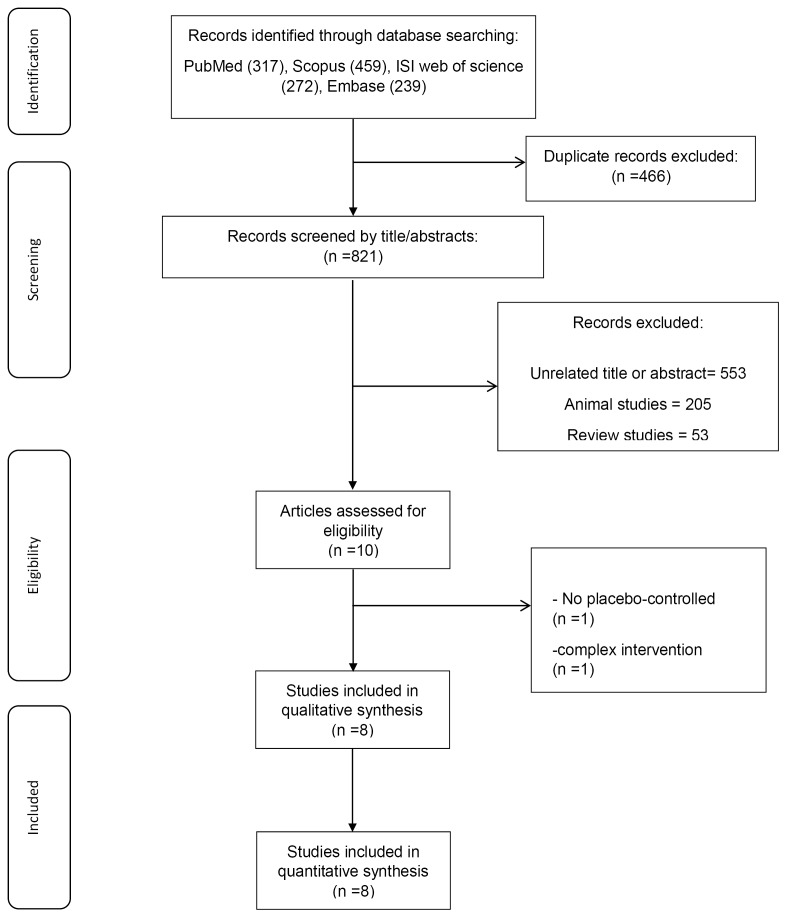
Flowchart of study selection for inclusion trials in the systematic review

In the first step, 1287 records were initially screened, and subsequently, we identified 466 duplication records, 205 animal model, 53 review articles, and 553 unrelated studies. After excluded these papers, 10 studies remained for full-text review. Two articles were excluded because of a lack of placebo/control group,^21^ and zinc supplementation in combination with another ingredient.^30^ Finally, 8 papers^16,17,22,25,31-34^ were eligible for this systematic review and meta-analysis. The search process is shown in Figure 1.

### Studies characteristics

The characteristics of the included studies are shown in Table 1. We obtained eight trials that evaluated effects of L-carnitine intervention on lipid profile in patients with T2D. These trials, involving 508 participants, were conducted in China,^31^ Iran,^17,33^ Italy,^16,25,32^ and Mexico.^22^ These participants age range was 44 to 61y, and a BMI ranging from 26.8 to 36 kg/m^2^. All of the included studies recruited both sexes.^16,17,22,25,31-34^ The range of the L-carnitine dosage was between 2000 mg/day to 3000 mg/day. The shortest intervention period was 4 weeks^22^ and the longest one was 52 weeks,^25^ whilst the smallest sample size was 12 participants^22^ and the largest sample size was 112.^34^

**Table 1:** Characteristics of included studies in systematic review and meta-analysis

**Author**	**Year**	**Country**	**Study design**	**Participants**	**Sex**	**Mean age (intervention/** **control)**	**Mean BMI (intervention/** **control)**	**Trial duration** **(wk)**	**Daily dose of L-carnitine (mg)**	**Sample size (intervention/control)**
Y Liang	1998	China	R/DB/PC	Patients with T2DM	F/M	59.4/57.9	27.2/26.9	12	3000	23/23
G Derosa	2003	Italy	R/DB/PC	Patients with T2DM	F/M	52/50	27.3/26.8	24	2000	46/48
AR Rahbar	2005	Iran	R/DB/PC	Patients with T2DM	F/M	50.5/52.2	27.9/28.2	12	3000	19/16
S Santo Signorelli	2006	Italy	R/DB/PC	Patients with T2DM	F/M	61.75/61.26	34/36	52	2000	37/37
M González-Ortiz	2008	Mexico	R/DB/PC	Patients with T2DM	F/M	44.1/42.6	27.5/27.8	4	3000	6/6
M Malaguarnera	2009	Italy	R/DB/PC	Patients with T2DM	F/M	49/48	27.5/27.4	12	2000	41/40
P Alavinejad	2016	Iran	R/DB/PC	Patients with T2DM	F/M	60/59	28.6/29.5	12	2250	28/26
A Parvanova	2018	Italy	R/DB/PC	Patients with T2DM	F/M	NR/NR	30.1/31	24	2000	55/57

Abbreviations: R, randomized; DB, double-blinded; PC, placebo-controlled; T2DM, type 2 diabetes mellitus; F, Female; M, Male; NR, not reported.

### Quality assessment

Random allocation of participants was indicated in all included studies.^16,17,22,25,31-34^ Allocation concealment of 5 trials^17,25,32-34^ was reported, whilst the remaining trials^16,22,31^ showed unclear risk of bias. Low risk of bias in all of the trials studies^16,17,22,25,31-34^ was indicated based on blinding of participants, personnel, and outcome assessment. Six studies^16,17,22,25,31,32^ showed low risk of bias based on incomplete outcome data and selective reporting. Seven trials illustrated low risk of bias based on selective outcome reporting.^16,17,22,25,31,32,34^ Details of risk of bias assessment are described in Table 2.

**Table 2:** Risk of bias for included studies

**Study**	**Random Sequence Generation**	**Allocation concealment**	**Blinding of participants personnel**	**Blinding of outcome assessors**	**Incomplete outcome data**	**Selective outcome reporting**	**Other sources of bias**
Y Liang	L	U	L	L	L	L	L
G Derosa	L	L	L	L	L	L	L
AR Rahbar	L	L	L	L	L	L	L
S Santo Signorelli	L	L	L	L	L	L	L
M González-Ortiz	L	U	L	L	L	L	L
M Malaguarnera	L	U	L	L	L	L	L
P Alavinejad	L	L	L	L	U	U	L
A Parvanova	L	L	L	L	U	L	L

### Effect of L-carnitine supplementation on blood total cholesterols

**Figure 2:**
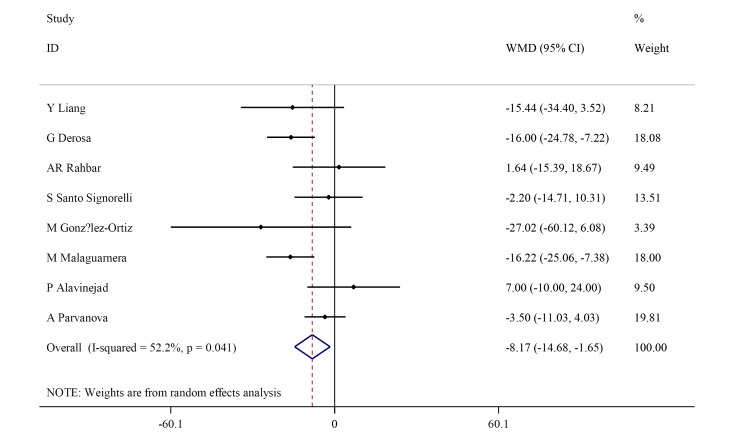
Forest plot detailing weighted mean difference and 95% confidence intervals (CIs) for the effect of L-carnitine supplementation on TC

This meta-analysis showed that L-carnitine supplement significantly decreased TC (WMD: -8.17 mg/dL; 95% CI, -14.68 to -1.65) with significant heterogeneity (I^2^=52.2%, *P* = 0.041) (Figure 2). After subgroup analysis, it was found that TC significantly decreased only in participants with high cholesterol (>200 mg/dL) (WMD: -11.93 mg/dL; 95% CI, -20.80 to -3.05). However, heterogeneity was not significant in participants with low cholesterol (<200 mg/dL) (I^2^=12.6%, *P* =0.319) or L-carnitine dosage ≥3000 mg/dL (I^2^=35.0%, *P* = 0.214) (Table 3).

**Table 3:** Subgroup analyses of l-carnitine supplementation on lipid profile

	**N**	**WMD (95%CI)**	***P*** ** within group**	***P*** ** heterogeneity**	**I** ^2^
**Subgroup analyses of l-carnitine supplementation on triglycerides level**					
Baseline serum triglycerides (mg/dL)					
>150	4	-9.52 (-51.40, 32.35)	0.656	<0.001	86.0%
<150	3	8.97 (-10.97, 28.92)	0.378	0.008	79.1%
Trial duration (week)					
≤12	5	-4.89 (-40.92, 31.13)	0.790	<0.001	81.7%
>12	2	8.03 (-14.48, 30.54)	0.484	0.002	89.5%
L-carnitine dose (mg)					
=3000	3	-8.70 (-115.38, 97.96)	0.873	<0.001	90.8%
<3000	4	2.64 (-11.92, 17.21)	0.722	<0.001	84.1%
**Subgroup analyses of l-carnitine supplementation on cholesterol level**					
Baseline serum cholesterol (mg/dL)					
>200	4	-11.93 (-20.80, -3.05)	0.008	0.098	52.4%
<200	3	-3.85 (-12.21, 4.50)	0.366	0.319	12.6%
Trial duration (week)					
≤12	5	-8.57 (-19.80, 2.65)	0.134	0.065	54.8%
>12	2	-9.53 (-21.78, 2.70)	0.127	0.034	77.7%
L-carnitine dose (mg)					
=3000	3	-10.01 (-25.40, 5.37)	0.202	0.214	35.0%
<3000	4	-8.70 (-17.77, 0.37)	0.060	0.016	71.1%
**Subgroup analyses of l-carnitine supplementation on HDL-C level**					
Baseline serum HDL-C (mg/dL)					
>40	5	-0.89 (-3.50, 1.71)	0.500	<0.001	88.1%
<40	1	-3.86 (-22.80, 15.08)	0.690	-	-
Trial duration (week)					
≤12	4	1.05 (0.32, 1.78)	0.005	0.508	0.0%
>12	2	-1.78 (-4.74, 1.18)	0.240	0.064	70.8%
L-carnitine dose (mg)					
=3000	3	-1.78 (-5.64, 2.07)	0.365	0.918	0.0%
<3000	3	-0.61 (-3.77, 2.55)	0.705	<0.001	93.9%
**Subgroup analyses of l-carnitine supplementation on LDL-C level**					
Baseline serum LDL-C (mg/dL)
>100	3	-9.26 (-15.04, -3.48)	0.002	0.197	38.5%
<100	2	8.33 (-17.19, 33.87)	0.522	0.023	80.8%
Trial duration (week)					
≤12	3	-4.43 (-28.83, 19.96)	0.721	0.005	81.3%
>12	2	-4.53 (-8.63, -0.44)	0.030	0.433	0.0%
Zinc dose (mg)					
=3000	2	-0.84 (-50.55, 48.86)	0.973	0.005	87.1%
<3000	3	-6.69 (-11.42, -1.96)	0.006	0.137	49.7%

### Effect of L-carnitine supplementation on blood HDL-cholesterols

**Figure 3:**
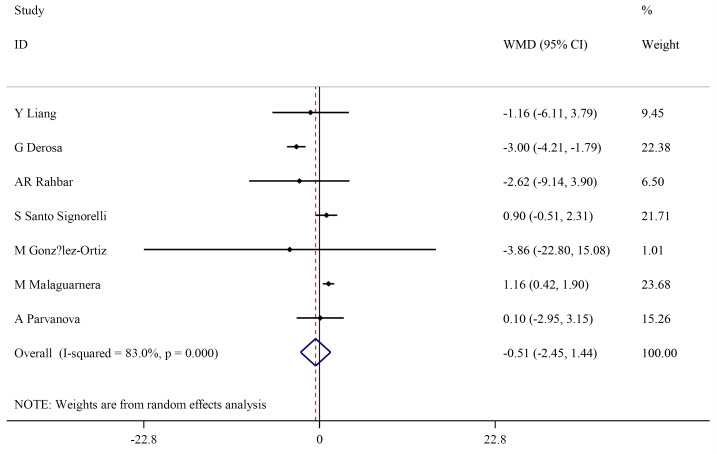
Forest plot detailing weighted mean difference and 95% confidence intervals (CIs) for the effect of carnitine supplementation on HDL-C

The serum level of HDL did not statistically change following L-carnitine intervention (WMD: -0.51 mg/dL; 95% CI, -2.45 to 1.44) (Figure 3) with significant heterogeneity among studies (I^2^=83.0%, *P* < 0.001). Subgroup analysis showed that L-carnitine supplementation does not show any significant effect on HDL serum level, except for trials with a duration ≤12 weeks. There was no significant heterogeneity in trials with a duration ≤12 weeks (I^2^=0.0%, *P* = 0.508) and L-carnitine dosages ≥3000 mg/dL (I^2^=0%, *P* = 0.918) (Table 3).

### Effect of L-carnitine supplementation on blood LDL-cholesterols

**Figure 4:**
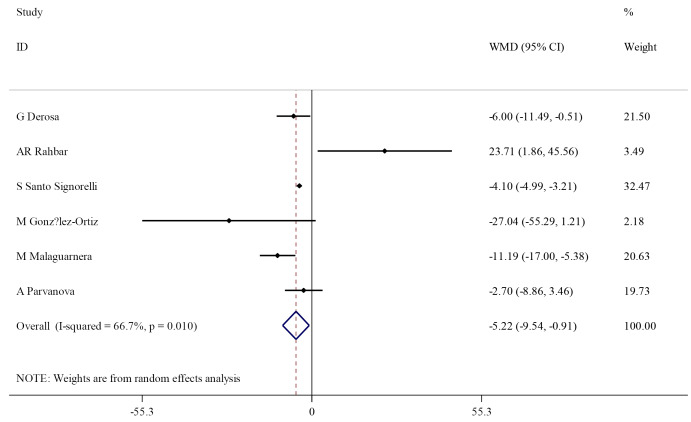
Forest plot detailing weighted mean difference and 95% confidence intervals (CIs) for the effect of carnitine supplementation on LDL-C

L-carnitine supplementation showed a significant effect on LDL (WMD: -5.22 mg/dL; 95% CI, -9.54 to -0.91), with significance heterogeneity (I^2^=66.7%, *P* = 0.010) (Figure 4). However, subgroup analysis showed that L-carnitine does significantly change serum LDL in subjects with low LDL level (<100 mg/dL), trial duration equal to or shorter than 12 weeks, and L-carnitine dosage ≥3000 mg/dL. On the other hand, heterogeneity was insignificant in subjects with high LDL (>100 mg/dL) (I^2^=38.5%, *P* = 0.197) and a trial duration longer than 12 weeks (I^2^=0.0%, *P* = 0.433) (Table 3).

### Effect of L-carnitine supplementation on blood triglycerides

**Figure 5:**
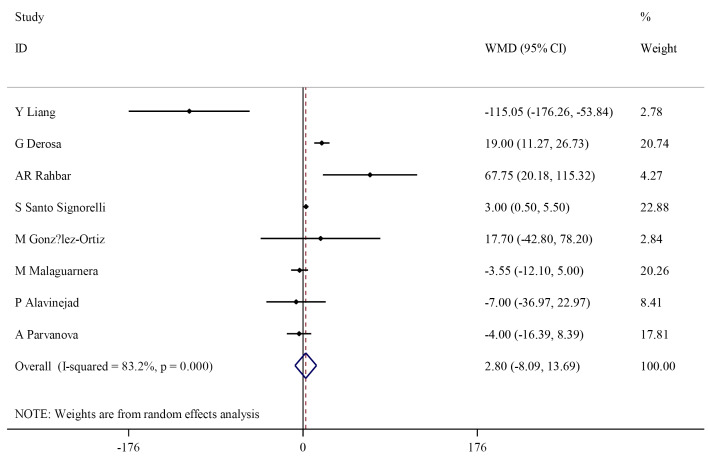
Forest plot detailing weighted mean difference and 95% confidence intervals (CIs) for the effect of carnitine supplementation on TG

Following L-carnitine intervention, TG level did not change significantly compared to control group (WMD: 2.80 mg/dL; 95% CI, -8.09 to 13.69), also, there was significant heterogeneity (I^2^=83.2%; *P* < 0.001) (Figure 5). However, the subgroup analysis showed that L-carnitine did not significantly effect TG serum level, and heterogeneity remained significant in all subgroups (Table 3).

### Publication bias and sensitivity analysis

In this meta-analysis, a possible source of publication bias was assessed by Egger’s test, and was not significant for TG (*P* = 0.351), TC (*P* = 0.259), HDL (*P* = 0.541) levels. However publication bias was evident for LDL (*P* = 0.18).

### Sensitivity analysis

A sensitivity analysis was carried out to evaluate the effectiveness of each single study on overall estimated effect size. The pooled effect size for TG was not significantly changed by excluding individual studies. However, the effect of L-carnitine on TC blood levels was significantly changed by removing the studies of Derosa et al^32^ (WMD: -6.44 mg/dL 95% CI: -13.52 to 0.64) and Malaguarnera et al^16^ (WMD: -6.40 mg/dL 95% CI: -13.43 to 0.62). Similarly, by eliminating two studies, Derosa et al^32^ and Malaguarnera et al,^16^ the effects of the L-carnitine on LDL were significantly changed and the results were (WMD: -4.86 mg/dL 95% CI: -10.71 to 0.98) and (WMD: -3.75 mg/dL 95% CI: -8.19 to 0.68), respectively. In the case of HDL, with the removal of one study,^36^ the results showed a significant increase in the level of HDL (WMD: 0.98 mg/dL 95% CI: 0.34 to 1.61).

## Discussion

This systematic review and meta-analysis showed that L-carnitine supplementation yielded a significant reduction in TC and LDL-C, without any significant change in serum levels of TG and HDL-C, in people with type 2 diabetes. According to our subgroup analysis, L-carnitine was more effective in reducing lipid concentrations in studies with an intervention duration of more than 12 weeks. Furthermore, L-carnitine in doses less than 3000 mg/d was more effective in altering TC and HDL-C. To the best of the author’s knowledge, this is the first meta-analysis to estimate the effect of L-carnitine supplementation on lipid concentration. Lipid abnormalities are characterized as high TC, high TG, low HDL-C, and increased levels of at least one marker are common in people with T2D and prediabetes,^35^ and epidemiologic evidence suggests that dyslipidemic profiles are independent risk factors for T2D.^36^ The third report of the National Cholesterol Education Program^37^ (NCEP) Expert Panel suggests that all of risk factors of patients with diabetes should be reduced in an effort to diminish the patients’ overall CVD risk. Therefore, finding a substance, natural or pharmacological, which could improve blood lipid abnormalities, especially in patients with T2D, is of great importance. Indeed, investigating the effect of nutraceuticals on cardiovascular risk factors represents a contemporary research topic.^38,39^ Several studies have reported the pharmacological effects of L-carnitine as an adjunctive therapy for dyslipidemia, particularly in patients with T2D.^17^ Animal studies have shown that high-dose L-carnitine treatment may reduce the severity of diabetes following an improvement in lipid profile and blood glucose.^40,41^ Moreover, intervention with L-carnitine in aged rodents showed a time-dependent normalization of abnormally elevated lipid peroxides and of subnormal antioxidant status.^42,43^ In addition, human studies have also demonstrated that L-carnitine concentrations in T2D patients with complications are 25% lower than those without complications, and it has been advocated that T2D patients may benefit from L-carnitine supplementation.^44,45^ A previous systematic review and meta-analysis, indicated that L-carnitine supplementation is associated to less mortality rate and a decrease in angina symptoms in patients with an acute myocardial infarction (AMI).^46^ In another meta-analysis, it was asserted that a dose of 3 grams per day L-carnitine is the dose necessitated to positively impact on all-cause mortality in AMI patients.^47^ Indeed, there was an improvement in TC and LDL-C levels following L-carnitine supplementation in our study. In line with our findings, a meta-analysis with only 4 studies showed that the intervention with L-carnitine is associated with a better glycemic status and lipid profile^48^; whilst another meta-analyses reported a marginal significant reduction of LDL-C, albeit in hemodialysis patients, although, they reported no significant change in TC.^49^

There is some evidence to suggest that in the gastrointestinal tract, due to the presence of gut microbiota, an unabsorbed part of l-carnitine is converted into trimethylamine (TMA), which repress reverse cholesterol transport and promotes the risk of arteriosclerotic vascular disease.^50^ Moreover, various studies have shown that the serum TMA might pertain to intakes from diet.^50-52^ According to the previous evidence that higher intake of red meat in compared to vegan intake could augment circulating TMAO after intake of L-carnitine^50^; on the other hand, however, the increasing acylcarnitines could be expressed as a risk factor for CVD, suggesting that L-carnitine might conceivably increase CVD risk through these pathways.

We also found a non-significant effect of L-carnitine on TG, which is concordant with previous meta-analyses.^48,53^ Several studies included in our study revealed that the reducing effect of L-carnitine on serum TG was obtained when L-carnitine were given to participants at doses of 2 g/d and 3 g/d, concurrently with a drug regimen,^16,19,21,54,55^ hypocaloric diet,^16,55^ or exercise.^56^ The route of L- carnitine intake might be influential on serum levels of L-carnitine and, likely, better L-carnitine bioaccumulation.^57^ However, it has been shown that acute L-carnitine treatment with a dose of 4 g/d for a week associated with fasting, had no reducing effects on TG levels, when compared to the control group,^13^ which could be attributed to the short trial durations. Moreover, it is noteworthy that the intracellular regulation of L-carnitine can be result of organic cation transporters (OCTNs) which are involved in the control of intestinal absorption, renal reabsorption, and tissue distribution of L-carnitine. Energy restriction, fasting state, and physical activity, can be associated with an elevated gene expression of OCTN2 in some tissues such as the liver and kidney; which in turn could lead to metabolism improvement.^14,58^ Furthermore, L-carnitine reportedly enhances the efficacy of fatty acid metabolism by increasing non-oxidative glucose disposal.^59^ Accordingly, L-carnitine supplementation can, conceivably, ameliorate the status of glycemic and lipid profile in patients with type 2 diabetes and metabolic syndrome.

This meta-analysis utilized randomized controlled trials with subgroup analysis for the dose and duration of L-carnitine supplementation, therein providing numerous strengths. Nevertheless, we had some limitations to this study that must be noted. First, the majority of included studies showed some form of bias, thus it is hard to yield a definitive conclusion. Second, we observed heterogeneity among the included studies, although the heterogeneity between studies was reduced by conducting subgroup analysis. Third, we could not control for the other potential covariates such as eating habits and lifestyle that can impact on the overall results.

## Conclusion

In conclusion, our findings indicated that L-carnitine supplementation may be effective in improving TC and LDL-C, particularly in doses less than 3000 mg/d. Further well-designed RCTs, with larger sample sizes are needed to better explicate the L-carnitine effect on people with type 2 diabetes.

## Competing interests

None to declared.

## Funding

None.
